# Managing cognition in progressive supranuclear palsy

**DOI:** 10.2217/nmt-2016-0027

**Published:** 2016-11-23

**Authors:** Timothy Rittman, Ian TS Coyle-Gilchrist, James B Rowe

**Affiliations:** 1 Department of Clinical Neurosciences, University of Cambridge, Cambridge, UK

**Keywords:** carer education, cognition, palliative care, PEG, progressive supranuclear palsy

## Abstract

Cognitive impairment is integral to the syndrome of progressive supranuclear palsy. It is most commonly described as a frontal dysexecutive syndrome but other impairments include apathy, impulsivity, visuospatial and memory functions. Cognitive dysfunction may be exacerbated by mood disturbance, medication and communication problems. In this review we advocate an individualized approach to managing cognitive impairment in progressive supranuclear palsy with the education of caregivers as a central component. Specific cognitive and behavioral treatments are complemented by treatment of mood disturbances, rationalizing medications and a patient-centered approach to communication. This aims to improve patients’ quality of life, reduce carer burden and assist people with progressive supranuclear palsy in decisions about their life and health, including discussions of feeding and end-of-life issues.

**Practice points**

**Cognitive dysfunction**

Cognitive impairment is integral to progressive supranuclear palsy (PSP).
The most common impairment is a frontal dysexecutive syndrome.
PSP may also affect memory, visuospatial function and emotional recognition.

**Behavioral changes**

Behavioral changes can occur early in PSP.
Apathy and impulsivity commonly co-exist in people with PSP.
Emotional lability may complicate the diagnosis of depression.

**Medication**

Side effects of levodopa can worsen cognition in PSP.
Amantadine can be helpful to improve some cognitive aspects including alertness and motivation, but may also cause hallucinations, insomnia and exacerbate impulsivity.
Anticholinergic medications to reduce saliva production and for urinary symptoms should be selected carefully to limit cognitive side effects.
Antidepressant medication should be chosen to limit side effects and antipsychotic medications should be avoided if at all possible.

**Educational & behavioral interventions**

There is limited evidence to guide specific interventions.
An individualized management plan is likely to be the optimum approach.
Carer education and support is important in managing the cognitive and behavioral aspects of PSP.

**Involving people with PSP in decisions**

Optimizing communication and cognition enables people with PSP to be involved in decisions about their life and health.
PEG tube insertion and end of life care are two common areas for discussion.
Early discussions and better integration with palliative care services can facilitate decision-making.

Progressive supranuclear palsy (PSP) usually affects cognitive function [1,2], despite the emphasis on motor deficits in the diagnostic criteria [3]. Cognitive impairment has a negative impact on the quality of life for people with PSP and their caregivers [4,5]. In this review we outline the main cognitive and behavioral changes encountered in PSP (see also Burrell *et al*. [2]) and consider the beneficial and detrimental effects of medications on cognition and behavior. We then present the evidence to support specific therapeutic intervention, and highlight the benefits of optimizing cognition to enable people with PSP to take an active role in decisions about their life and health.

The clinical syndrome of PSP was first described in 1964 [6], consisting of axial rigidity, akinesia, postural instability, pseudobulbar palsy, slow saccadic eye movements and a supranuclear gaze palsy [1,7,8]. In this review we will focus on the ‘Richardson syndrome’ or classical presentation of PSP. Other subtypes of PSP have been proposed based on differing initial presenting symptoms or respective review of post mortem cases of PSP. These subtypes include behavioral-onset PSP [9], Corticobasal Syndrome (CBS-PSP) [10], Progressive Akinesia with Gait Freezing (PAGF-PSP) [11,12] and Primary Progressive Aphasia (PPA-PSP) [13]. In addition, there is a substantial body of patients with neuropathological evidence of PSP but who clinically resembled Parkinson's disease (PD) ante mortem (PSP-P, a dopamine-responsive asymmetric syndrome [14]). The underlying pathology in these subtypes is difficult to predict, whereas the Richardson syndrome is associated with the pathological disease of PSP in over 90% of cases [15]. We, therefore, focus on the most common and typical Richardson syndrome of PSP, but because cognitive syndromes may overlap between PSP subtypes a similar approach to the concepts we discuss here may be applicable in other PSP syndromes.

The pathology of PSP is characterized by the accumulation of tau, a microtubule-associated protein. Hyperphosphorylated tau with four repeats of exon 10 form intracellular aggregates, including neurofibrillary tangles [16]. These tau aggregates are most densely deposited in the subthalamic nucleus, substantia nigra and internal globus pallidus, but are also formed widely in cerebellar and cortical regions [17]. Tau pathology is associated with rapid atrophy in subcortical structures and the midbrain [18,19] with moderate atrophy in frontal cortex [16]. This cortical and subcortical atrophy, and associated neurotransmitter deficits underlie the cognitive deficits of PSP.

## Cognitive dysfunction

The pattern of early cognitive change in PSP is typically summarized as a dysexecutive frontal syndrome [2,20,21]. This may manifest as difficulty with planning and organization and can be readily assessed at the bedside using tests such as the frontal assessment battery [22], verbal fluency [23] or neuropsychological tests such as Trail Making Test B or Brixton spatial anticipation test [24]. Slowness of cognitive processes, including slow speed of processing, is characteristic of PSP, and has been termed bradyphrenia.

Verbal fluency can be simply measured as the number of words generated in 1 min either within a category (e.g., animals) or beginning with a chosen letter (e.g., p-words). The striking deficit of letter fluency can aid the distinction of PSP from idiopathic PD; we have shown that patients who produced fewer than seven p-words in a minute were much more likely to have the syndrome of PSP rather than PD with a positive predictive value of this simple test alone of 0.81 and a negative predictive value of 0.93 [25]. Using the Frontal Assessment Battery and other tests of frontal lobe function, cognitive impairment is readily detectable at diagnosis but changes little over 12 months [24]. These findings suggest that frontal cognitive impairments are well established early in the disease process.

Frontal executive and verbal fluency changes are observed in most people with PSP, but other cognitive functions can be affected. Many people with PSP perform well on memory tests if they are given sufficient time to complete the test, but memory function can be affected [2] and may predict the level of disability better than other clinical features [26]. The mechanism of memory dysfunction may be related to frontal lobe pathology [21] distinct from the cholinergic mechanisms contributing to memory impairment in Alzheimer's disease. This hypothesis is consistent with the finding that cholinesterase inhibitors are not effective in PSP [27]. The domain of visuospatial function is often impaired in people with PSP [24,26], though may be overlooked in the context of other more striking changes.

Language deficits such as a progressive nonfluent aphasia (PNFA) may be the first presentation of PSP [13], or can appear later in the disease course. Changes of PNFA include agrammatism and impaired comprehension of syntactically complex sentence structure that may limit verbal interaction. Furthermore, communication may be affected by severe hypophonia and dysarthria in the later stages of disease.

Recently, a severe effect of PSP on emotional and social cognition has been recognized [28], not dissimilar to patients with another neurodegenerative tauopathy called frontotemporal dementia (FTD) [29]. This change in emotion recognition and understanding the intentions of other people will exacerbate the communication and behavioral problems that significantly contribute to the loss of quality of life for both patients and their carers.

## Behavioral changes

Changes in personality and behavior are often reported by carers, and confirmed by formal assessment tools such as the Cambridge Behavioral Inventory [30] or the Neuropsychiatric Inventory [31]. Behavioral changes may occur early in PSP, and a behavioral or ‘frontal’ presentation accounts for about 20% of all cases of PSP [9]. Behavioral changes are often apparent to families but are rarely reported by people with PSP themselves, a discrepancy that may be attributable to poor insight by patients [32].

In view of the akinesia (slowness of movement) in PSP, it surprises many carers and professionals that people with PSP are often also impulsive [33,34]. This may manifest as motor recklessness, such as getting up to walk despite the high chance of falling and after repeated warnings of the risks involved; or of cramming food into the mouth even while choking. Impulsivity may be accompanied by inflexibility and rigid thinking. Families may describe this trait as stubbornness, selfishness or ‘bloody-mindedness’, but it reflects damage to the brain circuits between the frontal lobe and basal ganglia that regulate goal-directed behavior and learning [35].

In parallel with this impulsive behavior is an almost universal loss of motivation and apathy [21] that reflects atrophy in medial frontal regions and insular cortex [36]. People with PSP tend not to initiate activities or conversations. This can be a source of frustration for families who may wish to keep their relative ‘active’ to preserve motor function, and can impair compliance with therapeutic interventions such as physiotherapy or speech exercises. In other neurodegenerative diseases apathy has been associated with increased carer distress [37–39], more rapid disease progression [40] and poorer prognosis. Reports of apathetic behavior from carers may be due to other factors. For example physical disability in PSP may lead to reduced spontaneous movement and goal directed behavior; facial hypomimia may be mistaken for lack of interest. Similarly, apathy may be mistaken for depression which may explain the marked differences between studies in the estimated rates of depression in PSP, ranging from 16% [21] to 56% [41]. However, while apathy and depression may coexist, they are distinct neuropsychological constructs [42]. Many apathetic patients will describe their own mood as good or happy and assert that they do still enjoy things. This contrasts with the perception of a lack of motivation and enjoyment observed by others. In our experience depression *per se* is relatively uncommon in PSP while apathy in the absence of persistent low mood is present in the majority of cases.

Contributing to these behaviors, the maintenance and recognition of emotions may also change in PSP. Emotional lability is a common symptom, characterized by relatively brief outbursts of emotion such as crying at a sad news story, or laughing inappropriately.

## Medication

The cognitive and behavioral features outlined above are a consequence of the underlying neuropathology in PSP, but may be exacerbated by treatments for the movement disorder and other symptoms of PSP. Levodopa and other dopaminergic medications are trialed in most people with PSP. In clear contrast to the sustained good response in PD dopaminergic medication is generally not effective in PSP, although may have a modest effect for a period of time in a minority of cases [43]. While the focus of most trials of dopaminergic therapy in PSP has been on the motor aspects of the disease, the cognitive effects of levodopa have mainly been studied in PD, demonstrating a nonlinear U-shaped association between cognitive function and dopaminergic medication that is influenced by the stage of disease [44,45]. While the effect (if any) of dopaminergic therapy on cognition in PSP is unclear, in PD smaller doses may improve performance on tasks of cognitive set shifting, but higher doses ‘overload’ the decision and motivation systems leading to impulsivity. The overload of these systems contributes to medication-related impulse control disorders which are relatively common in PD (10–15% [46]) compared with PSP, although there are a few case reports of dopamine-induced impulse control disorders in PSP [47].

Amantadine is also used in PD [48], with a variety of pharmacological actions, including NMDA antagonism [49]. Although there have been no clinical trials in PSP, we have often used amantadine in people with PSP, not only for its potential effect on akinetic rigidity but also to improve alertness, motivation, speech and balance. In a recent audit of cases, we assessed the effect of amantadine in 30 patients with PSP 57% self-reported one or more benefits from amantadine, with more likelihood of benefit in younger and milder patients [Yates T et al., Unpublished Data]. The benefits we found were related to motor function in nine subjects (two each with improved balance, speech, stiffness, tremor; one with walking), seven nonspecifically felt better and one had markedly reduced fatigue. Adverse reactions to amantadine were common but rapidly resolved on dose reduction: two patients developed hallucinations or aggressive behavior requiring early drug cessation; three patients, deriving no benefit, reported mild hallucinations and withdrew treatment; four developed hallucinations that resolved with reduced dosages and continued to benefit. Dose reduction abolished gastrointestinal disturbances in two cases, and insomnia and nonspecific unwellness in one case each. Mild livedo reticularis may also occur. In a single case study amantadine has been associated with myoclonus in PSP [50]. Our approach is to start on a low dose, 100 mg once daily, and escalate slowly over 2 months to a maximum dose of 200 mg twice daily or less according to tolerance, having carefully briefed the patient and carer about side effects.

Other common medications may have significant cognitive side effects, particularly the antimuscarinic medications used for incontinence, urgency and urinary frequency, and for other symptoms in PSP such as drooling (sometimes wrongly termed excessive saliva production). For example, Hyoscine is commonly used to reduce salivation but its systemic effects include confusion, unsteadiness and complaints of ‘muzzy-headedness’. Atropine drops used sublingually may be a better local treatment to reduce saliva production, with fewer systemic or cognitive side effects. There is limited evidence to support the use of trospium or darifenacin to treat bladder symptoms in people with neurodegenerative diseases as they cross the blood–brain barrier in smaller concentrations than alternatives [51,52], while noncholinergic approaches (e.g., Mirabegron) offer potential advantages.

There is a role for antidepressant medication in PSP to address mood disturbance which can affect cognition and behavior. However, before treatment of depression, one must ensure that the patient actually has a mood disorder. Apathy and facial hypomimia may falsely be interpreted as depression, while high scores on screening tests (e.g., the Beck Depression Inventory, the Hospital Anxiety and Depression Scale) may not reflect a mood disorder, but physical symptoms of PSP such as changes in sleep, weight, libido and fatigue. We suggest asking a patient specifically about their mood in concrete terms and closed questions, for example, ‘do you feel happy’ and ‘do you feel sad’. Although there is no clinical trials evidence to guide selection of any particular antidepressant in PSP, we often use citalopram for a rapid effect and low side effect profile. There is limited support for this approach from trials of selective serotonin reuptake inhibitors (SSRIs) in FTD where a systematic review and meta-analysis found that antidepressant treatment results in a 15.4 point reduction on the Neuropsychiatric Inventory with the strongest evidence for SSRIs, although acknowledging that the evidence was derived from small trials [53]. Furthermore, enhancing serotonergic transmission with citalopram may have an effect on response inhibition systems in FTD [54]. Trazadone is also well tolerated in people with neurodegenerative diseases [55,56] while Mirtazepine can promote sleep and the maintenance of weight which may be of benefit in PSP. A single case report suggests that repetitive transcranial magnetic stimulation is safe and may be effective for treatment-resistant depression in PSP [57]. Psychosis is rare in PSP, and there should be a high threshold for use of any antipsychotic medication given the evidence that long-term antipsychotic medication increases mortality in people with neurodegenerative disorders [58] and may worsen extrapyramidal syndromes.

There is a particular catch in the diagnosis of depression in PSP; the phenomenon of emotional lability or emotional incontinence. This causes patients to suddenly, and intensely cry and appear distressed, often for a short period (less than a minute). The patient's inner mood state may be very different from the external appearance. SSRIs are often effective for treating emotional incontinence, even in the absence of depression and often at lower doses than typically used to treat depression (e.g., citalopram 10 mg once daily).

Cholinesterase inhibitors and memantine have been successful medications for addressing cholinergic deficits in Alzheimer's disease, but have failed to show the same effect in PSP. An observational study of Rivastigmine in five people with PSP found a small improvement after 3–6 months in verbal fluency and backward digit span [59], but these results should be treated with caution since the study was small and unblinded, and there was a decline in all other cognitive assessments. There has been only a single randomized control trial of a cholinesterase inhibitor in PSP [27]. This trial assessed 21 patients over 6 weeks and found a deterioration in motor function and ability to carry out activities of daily living with only a small effect on memory.

Apathy is a particularly prominent cognitive feature of PSP and is a significant cause of patient morbidity [60,61] and (in other neurodegenerative conditions) has been linked to significant carer distress [62]. This makes it an attractive target for symptomatic therapy; however, evidence for particular pharmacological treatments of apathy in PSP is limited. In a double-blind randomized placebo controlled trial the GSK-3 inhibitor tideglusib had no effect on apathy (measured by the Straskiein scale) among patients with PSP at 52 weeks [63]. In other conditions, there is limited evidence to suggest modest effects of cholinesterase inhibitors improve apathetic symptoms in dementia [64,65], PD [66] and traumatic brain injury [67]. Dopamine agonists have been used following stroke [68] and in case reports amantadine [69] and selegiline [70]. The stimulant modafinil showed some improvement in apathy in Alzheimer's disease in a double blind placebo controlled trial [71] but another randomized trial showed no effect [72]. Dramatic improvement in the symptoms of PSP (including apathy) has been reported with the GABA agonist zolpidem in an individual case [73] but further evidence is lacking. Because apathy is not a single unitary construct but reflects multiple neuropsychological processes associated with disruption of a number of prefrontal and basal ganglia circuits [74] it is not clear which (if any) of these interventions are likely to be effective in the treatment of apathy in PSP.

Recent efforts to find a disease-modifying treatments for PSP have focused on disrupting the aggregation of hyperphosphorylated tau species. Trials have failed to show any significant effect on disease progression, including cognitive measures, for sodium valproate [75], davunetide [76], tideglusib [63] or riluzole [15]. However, a series of new disease-modifying treatment trials are under way or planned, including immunotherapies. Within 5 years, the scope for disease modification may be much improved.

In the recent years, rasagaline, a monoamine oxidase inhibitor, is suggested to have a disease modifying effect in idiopathic PD in the ADAGIO trial, although a positive result was seen only in the lower dose arm of the study [77]. A trial of rasagaline in 44 patients with PSP showed no effect on disease progression measured by the PSP Rating Scale [78].

## Educational & behavioral interventions

Given the limitations of medication in managing cognitive and behavioral symptoms in PSP, nonpharmacological interventions should be the first line of treatment. These interventions are likely to have few side effects, but may require additional personnel and resources to implement, including education and support for caregivers. Confidence in the diagnosis, and education about the illness can in itself make a significant contribution. Understanding that personality and behavioral change are a result of PSP and not intentional callousness or selfishness can go a long way to reducing the problems they cause.

Limited evidence exists for nonpharmacological interventions across different neurodegenerative disorders [79]. In PSP, evidence supporting general rehabilitation approaches are limited to a few case studies [80], and we are unaware of any published interventions specifically targeting cognition in PSP. A study providing individual assessment for 153 people with Alzheimer's disease followed by a considered intervention reduced behavioral symptoms and carer distress with follow-up over 18 months [81]. Interventions included cholinesterase inhibitors or memantine, communication skills education, caregiver coping skills, legal and finance advice, exercise and a caregiver guide. The study team assigned a specialist nurse embedded in a multidisciplinary team and in close partnership with caregivers. Such a framework is likely to be the optimum approach across neurodegenerative disorders, although crucially the interventions did not improve scores on cognitive tests. Despite this caveat, we believe that the end point of any intervention should be relevant to the quality of life rather than aiming to improve cognition and therefore the measurement of cognitive scores should be a secondary consideration when planning or evaluating such a study.

Translating individual interventions between different neurodegenerative diseases can be problematic. For example, disorientation and memory symptoms that are common in Alzheimer's disease are less prominent in PSP, and impairment of motivation, movement and communication may limit people with PSP participating in activities that may be of benefit in other types of dementia.

The assessment of cognition and behavior should include multiple sources of reference. Clinicians and formal assessment tools may be useful to identify underlying cognitive changes but may not easily translate into meaningful outcomes in the real world. Patients often lack insight into their own behavior and disability; carers, despite being best placed to report problematic behaviors in the day-to-day setting, may also misinterpret the patient's motivation or behavior, or project their own preconceptions or distress on the patient.

There is emerging evidence to guide the support of caregivers, although clinical trials are needed. A telehealth intervention has been proposed to educate caregivers of people with PSP, although this was not particularly targeted at cognition and its efficacy has yet to be established [82]. A review of psychological input for family caregivers in other neurodegenerative diseases found mainly poor quality studies, but some evidence to support interventional Behavioral Management Therapy at an individual level rather than in a group environment [83]. These findings further support the benefit of individualized, tailored interventions for people with PSP and their carers. In this review, there was no evidence to support the education of caregivers alone. However, rarer forms of dementia such as PSP might be a special case. Specific education has been highlighted as a need for carers of people with FTD in comparison to those caring for people with Alzheimer's disease [84,85].

We propose a set of general principles for approaching behavioral disturbance in PSP (Box 1). These principles recognize the unique behavioral challenges in PSP while drawing on aspects neurodegenerative disorders more generally. Further studies are urgently needed to provide guidance in managing specific behavioral symptoms to guide caregivers and professionals in this difficult area.

## Involving people with PSP in decisions

An important goal of maximizing cognition and communication is to involve people with PSP in decisions about their life and healthcare. To demonstrate the importance of improving cognition in PSP, we will discuss two areas for decision-making with which medical teams are often involved: inserting a percutaneous endoscopic gastrostomy (PEG) feeding tube; and end of life decisions.

Considering a PEG in people with PSP is common since swallowing problems usually develop during the course of PSP, leading to a risk of aspiration pneumonia, weight loss, unpleasant choking episodes and lengthy mealtimes. Although a PEG tube does not prevent aspiration pneumonia, it can have benefits in terms of maintaining nutrition and comfort and does not preclude eating for pleasure. There is a lack of evidence to help with the timing of PEG placement, although studies in motor neuron disease support early placement since waiting until there is significant weight loss, malnutrition and dysphagia increases postprocedure mortality [86], though practice varies between centers on optimal timing for PEG placement. Many people with PSP do not wish to have a PEG tube for cosmetic reasons, concerns about the insertion procedure or because they do not like the idea of ‘artificial feeding’. Patients may also misunderstand the palliative role of PEG, rather than prolonging life *per se*.

Similarly, discussions around end of life care are sensitive and often very personal. These include decisions about where to die, whether to be admitted to hospital to treat infections and resuscitation status. Poor communication between neurologists and palliative care teams may hinder such discussions [87] and a recent survey across the UK highlights the variability between regions in the integration of neurology and palliative care services in PSP and other neurological disorders [88]. We advocate early discussions about palliative care issues and early involvement of palliative care teams to assist with advance care planning in PSP. It may not be comfortable for the clinician to talk about death, fear of death, the processes of dying, and guilt about surviving carers, but patients may be preoccupied and worried by these issues. Clarifying their concerns reduces the risk of making false assumptions and can enable reassurance and practical measures to be put in place.

In the early years of PSP around the time of diagnosis, the majority of people with PSP will have the communication and cognitive abilities to discuss PEG placement and end of life decisions. Later on in the disease this legal process may need facilitating by a careful assessment of capacity, maximizing cognitive function and optimizing communication.

To properly involve people with PSP and impaired cognition in these important decisions takes time and patience. A discussion that may take a few minutes with a healthy person can take half an hour in someone with PSP. Our experience is that this is time well spent, and a clearly documented discussion can pave the way for clear decision-making in moments of crisis. There is some evidence that Lee Silverman exercises, designed to address hypophonia in idiopathic PD, can be of benefit in promoting communication in PSP [89]. Further specific guidance for improving communication in PSP is greatly needed but currently lacking [90]. In addition to the medical and nonmedical measures to improve cognition outlined in preceding sections, the environment in which a decision is made can be optimized to assist decision-making [91].

Supporting these decisions within a legal framework can add an additional level of protection for an individual's wishes [92]. In the UK the Mental Capacity Act 2005 provides the framework within which these decisions are made, although the implementation of the Act by neurologists is variable [93]. The Mental Capacity Act makes provision to appoint a lasting power of attorney, whereby a person of sound mind can nominate someone to take on financial and medical decisions if they become incapacitated at a later date. In addition, an individual can make more specific statements to refuse care, given a certain set of circumstances written down as advanced statements, or more formal legal documents called advance directives.

## Future perspective

In 5–10 year's time we hope that cognitive dysfunction in PSP will be recognized as a core feature in revised diagnostic guidelines. We would like to see the standard evaluation of a person with PSP to include a formal assessment of cognition, and the standard treatment to begin with formalized education of carers about cognitive issues. We expect that increased awareness of the Mental Health Act and the growth of neuropalliative care will lead to better integration between neurologists and palliative care physicians enabling better optimization of cognition and communication to facilitate advanced care planning and avoiding crisis situations.

## Conclusion

In conclusion, the Richardson syndrome of PSP is associated with many cognitive and behavioral changes, including apathy, impulsivity and a frontal dysexecutive syndrome. Cognition can be worsened by side effects of common medications. There is little clinical trials’ evidence to guide the management of these symptoms, but here we set out the rationale and strategies for treating mood disturbance, educating patients and their caregivers, and the benefits of involving people with PSP in decision-making about their life and health.

**Box 1.**  **Principles of behavioral management in progressive supranuclear palsy.**

Gather information on behavior from multiple sources, for example the patient, family, professional caregivers, therapists
Specific education for caregivers about the cognitive and behavioral spectrum of progressive supranuclear palsy, particularly apathy, impulsivity and emotional lability
Address systemic causes of behavioral change, for example infection, constipation, pain
Reduce medication where possible that may cause cognitive impairment, drowsiness or hallucinations
Low-dose selective serotonin reuptake inhibitor may help to treat emotional lability

## References

[1] Litvan I, Mangone CA, McKee A (1996). Natural history of progressive supranuclear palsy (Steele-Richardson-Olszewski syndrome) and clinical predictors of survival: a clinicopathological study. *J. Neurol. Neurosurg. Psychiatry*.

[2] Burrell JR, Hodges JR, Rowe JB (2014). Cognition in corticobasal syndrome and progressive supranuclear palsy: a review. *Mov. Disord.*.

[3] Litvan I, Agid Y, Calne D (1996). Clinical research criteria for the diagnosis of progressive supranuclear palsy (Steele-Richardson-Olszewski syndrome): report of the NINDS-SPSP international workshop. *Neurology*.

[4] Uttl B, Santacruz P, Litvan I, Grafman J (1998). Caregiving in progressive supranuclear palsy. *Neurology*.

[5] Pekmezovic T, Jecmenica-Lukic M, Petrovic I, Spica V, Tomic A, Kostic VS (2015). Quality of life in patients with progressive supranuclear palsy: one-year follow-up. *J. Neurol.*.

[6] Steele JC, Richardson JC, Olszewski J (1964). Progressive supranuclear palsy: a heterogenous degeneration involving the brain stem, basal ganglia and cerebellum with vertical gaze and pseudobulbar palsy, nuchal dystonia and dementia. *Arch. Neurol.*.

[7] Probst A, Langui D, Lautenschlager C (1988). Progressive supranuclear palsy: extensive neuropil threads in addition to neurofibrillary tangles. *Acta Neuropathol.*.

[8] Komori T (1999). Tau-positive glial inclusions in progressive supranuclear palsy, corticobasal degeneration and Pick's disease. *Brain Pathol.*.

[9] Kaat LD, Boon AJW, Kamphorst W, Ravid R, Duivenvoorden HJ, Van Swieten JC (2007). Frontal presentation in progressive supranuclear palsy. *Neurology*.

[10] Armstrong MJ, Litvan I, Lang AE (2013). Criteria for the diagnosis of corticobasal degeneration. *Neurology*.

[11] Factor S, Higgins D (2006). Primary progressive freezing gait: a syndrome with many causes. *Neurology*.

[12] Williams DR, Holton JL, Strand K, Revesz T, Lees AJ (2007). Pure akinesia with gait freezing: a third clinical phenotype of progressive supranuclear palsy. *Mov. Disord.*.

[13] Rohrer JD, Paviour DC, Bronstein AM, O'Sullivan SS, Lees A, Warren JD (2010). Progressive supranuclear palsy syndrome presenting as progressive nonfluent aphasia: a neuropsychological and neuroimaging analysis. *Mov. Disord.*.

[14] Williams DR, de Silva R, Paviour DC (2005). Characteristics of two distinct clinical phenotypes in pathologically proven progressive supranuclear palsy: Richardson's syndrome and PSP-parkinsonism. *Brain*.

[15] Bensimon G, Ludolph A, Agid Y, Vidailhet M, Payan C, Leigh PN (2009). Riluzole treatment, survival and diagnostic criteria in Parkinson plus disorders: the NNIPPS study. *Brain*.

[16] Dickson DW, Rademakers R, Hutton ML (2007). Progressive supranuclear palsy: pathology and genetics. *Brain Pathol.*.

[17] Williams DR, Lees AJ (2009). Progressive supranuclear palsy: clinicopathological concepts and diagnostic challenges. *Lancet Neurol.*.

[18] Paviour DC, Price SL, Jahanshahi M, Lees AJ, Fox NC (2006). Longitudinal MRI in progressive supranuclear palsy and multiple system atrophy: rates and regions of atrophy. *Brain*.

[19] Ghosh BCP, Calder AJ, Peers PV (2012). Social cognitive deficits and their neural correlates in progressive supranuclear palsy. *Brain*.

[20] Gerstenecker A, Mast B, Duff K, Ferman TJ, Litvan I (2012). Executive dysfunction is the primary cognitive impairment in progressive supranuclear palsy. *Arch. Clin. Neuropsychol.*.

[21] Millar D, Griffiths P, Zermansky AJ, Burn DJ (2006). Characterizing behavioral and cognitive dysexecutive changes in progressive supranuclear palsy. *Mov. Disord.*.

[22] Dubois B, Slachevsky A, Litvan I, Pillon B (2000). The FAB: A frontal assessment battery at bedside. *Neurology*.

[23] Bak TH, Crawford LM, Hearn VC, Mathuranath PS, Hodges JR (2005). Subcortical dementia revisited: similarities and differences in cognitive function between progressive supranuclear palsy (PSP), corticobasal degeneration (CBD) and multiple system atrophy (MSA). *Neurocase*.

[24] Ghosh BCP, Carpenter RHS, Rowe JB (2013). A longitudinal study of motor, oculomotor and cognitive function in progressive supranuclear palsy. *PLoS ONE*.

[25] Rittman T, Ghosh BCP, McColgan P (2013). The Addenbrooke's Cognitive Examination for the differential diagnosis and longitudinal assessment of patients with parkinsonian disorders. *J. Neurol. Neurosurg. Psychiatry*.

[26] Cushing N, Jang J, O'Connor CM (2013). Disability in atypical parkinsonian syndromes is more dependent on memory dysfunction than motor symptoms. *Parkinsonism Relat. Disord.*.

[27] Litvan I, Phipps M, Pharr VL, Hallett M, Grafman J, Salazar A (2001). Randomized placebo-controlled trial of donepezil in patients with progressive supranuclear palsy. *Neurology*.

[28] Ghosh BCP, Rowe JB, Calder AJ, Hodges JR, Bak TH (2009). Emotion recognition in progressive supranuclear palsy. *J. Neurol. Neurosurg. Psychiatry*.

[29] Lagarde J, Valabrègue R, Corvol J-C (2013). Are frontal cognitive and atrophy patterns different in PSP and bvFTD? A comparative neuropsychological and VBM Study. *PLoS ONE*.

[30] Wedderburn C, Wear H, Brown J (2008). The utility of the Cambridge Behavioural Inventory in neurodegenerative disease. *J. Neurol. Neurosurg. Psychiatry*.

[31] Cummings JL, Mega M, Gray K, Rosenberg-Thompson S, Carusi DA, Gornbein J (1994). The neuropsychiatric inventory: comprehensive assessment of psychopathology in dementia. *Neurology*.

[32] O'Keeffe FM, Murray B, Coen RF (2007). Loss of insight in frontotemporal dementia, corticobasal degeneration and progressive supranuclear palsy. *Brain*.

[33] Gerstenecker A, Duff K, Mast B, Litvan I (2013). Behavioral abnormalities in progressive supranuclear palsy. *Psychiatry Res.*.

[34] Zhang J, Rittman T, Nombela C (2016). Different decision deficits impair response inhibition in progressive supranuclear palsy and Parkinson's disease. *Brain*.

[35] Rowe JB, Rittman T, Hussain M, Schott JM (2016). The basal ganglia in cognitive disorders. *Oxford Textbook of Cognitive Neurology and Dementia*.

[36] Stanton BR, Leigh PN, Howard RJ, Barker GJ, Brown RG (2013). Behavioural and emotional symptoms of apathy are associated with distinct patterns of brain atrophy in neurodegenerative disorders. *J. Neurol.*.

[37] Oh Y-S, Lee JE, Lee PH, Kim J-S (2015). Neuropsychiatric symptoms in Parkinson's disease dementia are associated with increased caregiver burden. *J. Mov. Disord.*.

[38] Diehl-Schmid J, Schmidt E-M, Nunnemann S (2013). Caregiver burden and needs in frontotemporal dementia. *J. Geriatr. Psychiatry Neurol.*.

[39] Conde-Sala JL, Garre-Olmo J, Turró-Garriga O, Vilalta-Franch J, López-Pousa S (2010). Differential features of burden between spouse and adult-child caregivers of patients with Alzheimer's disease: an exploratory comparative design. *Int. J. Nurs. Stud.*.

[40] Richard E, Schmand B, Eikelenboom P (2012). Symptoms of apathy are associated with progression from mild cognitive impairment to Alzheimer's disease in non-depressed subjects. *Dement. Geriatr. Cogn. Disord.*.

[41] Schrag A, Sheikh S, Quinn NP (2010). A comparison of depression, anxiety, and health status in patients with progressive supranuclear palsy and multiple system atrophy. *Mov. Disord.*.

[42] Levy ML, Cummings JL, Fairbanks LA (1998). Apathy is not depression. *J. Neuropsychiatry Clin. Neurosci.*.

[43] Kompoliti K, Goetz CG, Litvan I, Jellinger K, Verny M (1998). Pharmacological therapy in progressive supranuclear palsy. *Arch. Neurol.*.

[44] Rowe JB, Hughes LE, Ghosh BCP (2008). Parkinson's disease and dopaminergic therapy – differential effects on movement, reward and cognition. *Brain*.

[45] Gotham AM, Brown RG, Marsden CD (1988). ‘Frontal’ cognitive function in patients with parkinson's disease ‘on’ and ‘off’ levodopa. *Brain*.

[46] Weintraub D, Potenza M (2010). Impulse control disorders in Parkinson's disease: a cross-sectional study of 3090 patients. *Arch. Neurol.*.

[47] O'Sullivan SS, Djamshidian A, Ahmed Z (2010). Impulsive-compulsive spectrum behaviors in pathologically confirmed progressive supranuclear palsy. *Mov. Disord.*.

[48] Schwab RS (1969). Amantadine in the treatment of Parkinson's disease. *JAMA*.

[49] Brichta L, Greengard P, Flajolet M (2013). Advances in the pharmacological treatment of Parkinson's disease: targeting neurotransmitter systems. *Trends Neurosci.*.

[50] Yarnall AJ, Burn DJ (2012). Amantadine-induced myoclonus in a patient with progressive supranuclear palsy. *Age Ageing*.

[51] Staskin D, Kay G, Tannenbaum C (2010). Trospium chloride has no effect on memory testing and is assay undetectable in the central nervous system of older patients with overactive bladder. *Int. J. Clin. Pract.*.

[52] Kay GG, Ebinger U (2008). Preserving cognitive function for patients with overactive bladder: evidence for a differential effect with darifenacin. *Int. J. Clin. Pract.*.

[53] Huey ED, Putnam KT, Grafman J (2015). A systematic review of neurotransmitter deficits and treatments in frontotemporal dementia. *Neurology*.

[54] Hughes LE, Rittman T, Regenthal R, Robbins TW, Rowe JB (2015). Improving response inhibition systems in frontotemporal dementia with citalopram. *Brain*.

[55] Houlihan DJ, Mulsant BH, Sweet RA (1994). A naturalistic study of trazodone in the treatment of behavioral complications of dementia. *Am. J. Geriatr. Psychiatry*.

[56] Sultzer DL, Gray KF, Gunay I, Berisford MA, Mahler ME (1997). A double-blind comparison of trazodone and haloperidol for treatment of agitation in patients with dementia. *Am. J. Geriatr. Psychiatry*.

[57] Boulogne S, Le Camus F, Bation R (2015). Repetitive transcranial magnetic stimulation can alleviate treatment-resistant depression in patients with progressive supranuclear palsy. *Parkinsonism Relat. Disord.*.

[58] Ballard C, Hanney ML, Theodoulou M (2009). The dementia antipsychotic withdrawal trial (DART-AD): long-term follow-up of a randomised placebo-controlled trial. *Lancet Neurol.*.

[59] Liepelt I, Gaenslen A, Godau J (2010). Rivastigmine for the treatment of dementia in patients with progressive supranuclear palsy: clinical observations as a basis for power calculations and safety analysis. *Alzheimers Dement.*.

[60] Bak TH, Crawford LM, Berrios G, Hodges JR (2010). Behavioural symptoms in progressive supranuclear palsy and frontotemporal dementia. *J. Neurol. Neurosurg. Psychiatry*.

[61] Brown RG, Lacomblez L, Landwehrmeyer BG (2010). Cognitive impairment in patients with multiple system atrophy and progressive supranuclear palsy. *Brain*.

[62] Leiknes I, Tysnes O-B, Aarsland D, Larsen JP (2010). Caregiver distress associated with neuropsychiatric problems in patients with early Parkinson's disease: the Norwegian ParkWest Study. *Acta Neurol. Scand.*.

[63] Tolosa E, Litvan I, Höglinger GU (2014). A Phase 2 trial of the GSK-3 inhibitor tideglusib in progressive supranuclear palsy. *Mov. Disord.*.

[64] Trinh N-H, Hoblyn J, Mohanty S, Yaffe K (2003). Efficacy of cholinesterase inhibitors in the treatment of neuropsychiatric symptoms and functional impairment in Alzheimer disease. *JAMA*.

[65] Marin RS, Wilkosz PA (2005). Disorders of diminished motivation focus on clinical practice and research. *J. Head Trauma Rehabil.*.

[66] Devos D, Moreau C, Maltête D (2014). Rivastigmine in apathetic but dementia and depression-free patients with Parkinson's disease: a double-blind, placebo-controlled, randomised clinical trial. *J. Neurol. Neurosurg. Psychiatr.*.

[67] Masanic Ca, Bayley MT, Van Reekum R, Simard M (2001). Open-label study of donepezil in traumatic brain injury. *Arch. Phys. Med. Rehabil.*.

[68] Adam R, Leff A, Sinha N (2013). Dopamine reverses reward insensitivity in apathy following globus pallidus lesions. *Cortex*.

[69] Van Reekum R, Bayley M, Garner S (1995). N of 1 study: amantadine for the amotivational syndrome in a patient with traumatic brain injury. *Brain Inj.*.

[70] Newburn G, Newburn D (2005). Selegiline in the management of apathy following traumatic brain injury. *Brain Inj.*.

[71] Rosenberg PPB, Lanctot KL, Drye LT (2013). Safety and efficacy of methylphenidate for apathy in Alzheimer's disease: a randomized, placebo-controlled trial. *J. Clin. Psychiatry*.

[72] Frakey LL, Salloway S, Buelow M, Malloy P (2012). A randomized, double-blind, placebo-controlled trial of modafinil for the treatment of apathy in individuals with mild-to-moderate Alzheimer's disease. *J. Clin. Psychiatry*.

[73] Cotter C, Armytage T, Crimmins D (2010). The use of zolpidem in the treatment of progressive supranuclear palsy. *J. Clin. Neurosci.*.

[74] Levy R, Dubois B (2006). Apathy and the functional anatomy of the prefrontal cortex-basal ganglia circuits. *Cereb. Cortex*.

[75] Leclair-Visonneau L, Rouaud T, Debilly B (2016). Randomized placebo-controlled trial of sodium valproate in progressive supranuclear palsy. *Clin. Neurol. Neurosurg.*.

[76] Boxer AL, Lang AE, Grossman M (2014). Davunetide in patients with progressive supranuclear palsy: a randomised, double-blind, placebo-controlled Phase 2/3 trial. *Lancet Neurol.*.

[77] Olanow CW, Rascol O, Hauser R (2009). A double-blind, delayed-start trial of rasagiline in Parkinson's disease. *N. Engl. J. Med.*.

[78] Nuebling G, Hensler M, Paul S, Zwergal A, Crispin A, Lorenzl S (2016). PROSPERA: a randomized, controlled trial evaluating rasagiline in progressive supranuclear palsy. *J. Neurol.*.

[79] Douglas S, James I, Ballard C (2004). Non-pharmacological interventions in dementia non-pharmacological interventions in dementia. *Adv. Psychiatr. Treat.*.

[80] Golbe L (2014). Progressive supranuclear palsy: disease profile and rehabilitation strategies. *Semin Neurol.*.

[81] Callahan CM, Boustani MA, Unverzagt FW (2006). Effectiveness of collaborative care for older adults with Alzheimer disease in primary care: a randomized controlled trial. *JAMA*.

[82] Dunlop SR, Kent VP, Lashley M, Caruana T (2016). The Cure PSP Care Guide: a telephonic nursing intervention for individuals and families living with progressive supranuclear palsy. *J. Neurosci. Nurs.*.

[83] Selwood A, Johnston K, Katona C, Lyketsos C, Livingston G (2007). Systematic review of the effect of psychological interventions on family caregivers of people with dementia. *J. Affect. Disord.*.

[84] Nicolaou PL, Egan SJ, Gasson N, Kane RT (2010). Identifying needs, burden, and distress of carers of people with frontotemporal dementia compared with Alzheimer's disease. *Dementia*.

[85] Rosness TA, Haugen PK, Engedal K (2008). Support to family carers of patients with frontotemporal dementia. *Aging Ment. Heal.*.

[86] Rio A, Ellis C, Shaw C (2010). Nutritional factors associated with survival following enteral tube feeding in patients with motor neuron disease. *J. Hum. Nutr. Diet.*.

[87] Oliver DJ, Borasio GD, Caraceni A (2015). A consensus review on the development of palliative care for patients with chronic and progressive neurological disease. *Eur. J. Neurol.*.

[88] van Vliet LM, Gao W, DiFrancesco D (2016). How integrated are neurology and palliative care services? Results of a multicentre mapping exercise. *BMC Neurol.*.

[89] Sale P, Castiglioni D, De Pandis MF (2015). The Lee Silverman Voice Treatment (LSVT^®^) speech therapy in progressive supranuclear palsy. *Eur. J. Phys. Rehabil. Med.*.

[90] Kim JH, McCann CM (2015). Communication impairments in people with progressive supranuclear palsy: a tutorial. *J. Commun. Disord.*.

[91] Ball LJ, Beukelman DR, Pattee GL (2004). Communication effectiveness of individuals with amyotrophic lateral sclerosis. *J. Commun. Disord.*.

[92] Kerrigan S, Ormerod I (2010). Advance planning in end-of-life care: legal and ethical considerations for neurologists. *Pract. Neurol.*.

[93] Wilson E, Seymour JE, Perkins P (2010). Working with the Mental Capacity Act: findings from specialist palliative and neurological care settings. *Palliat. Med.*.

